# Process Intensification of Tetracycline Degradation: Synergistic Electrocatalytic Ozonation and Photoelectrocatalysis in Aqueous and Organic Matrices

**DOI:** 10.1021/acsomega.6c04336

**Published:** 2026-06-30

**Authors:** William Santacruz, Michel Z. Fidelis, Julia Faria, Artur J. Motheo

**Affiliations:** São Carlos Institute of Chemistry, 153988 University of São Paulo (USP), São Carlos, SP CEP 13560-97, Brazil

## Abstract

The persistence of antibiotics in wastewater necessitates advanced treatment strategies to reduce environmental risk. This study investigates the degradation of tetracycline (TC) using a Ti_0.7_Ru_0.3_O_2_-mixed metal oxide (MMO) anode through electrocatalysis (EC), photocatalysis (PC), ozonation (Oz), and their coupled configurations: photoelectrocatalysis (PEC) and electrocatalytic ozonation (ECOz). Water, methanol, and ethanol were used to evaluate solvent-dependent radical generation and degradation pathways in concentrated antibiotic effluents, simulating real-world postadsorption regeneration scenarios. EC achieved >98% removal in water and methanol but was limited in ethanol (92.5%) due to stable carbon-centered radical formation. Ozonation was highly effective in water (99.3%) but failed in organic media (<8%). In contrast, coupled processes (PEC and ECOz) consistently achieved ∼99% removal across all solvents. Notably, ECOz in water exhibited the highest synergy index (SI = 4.76). Mechanistic analysis via LC-MS identified key intermediates, revealing that while single processes often generated persistent and potentially more toxic byproducts, coupled systems promoted deeper oxidation toward mineralization. Effective energy consumption (EEC) calculations showed that process integration significantly improved kinetic rates, offsetting the additional power requirements. These findings demonstrate that integrating electrochemical AOPs is a robust strategy for treating concentrated antibiotic streams, bridging the gap between pollutant separation and safe environmental discharge.

## Graphical abstract

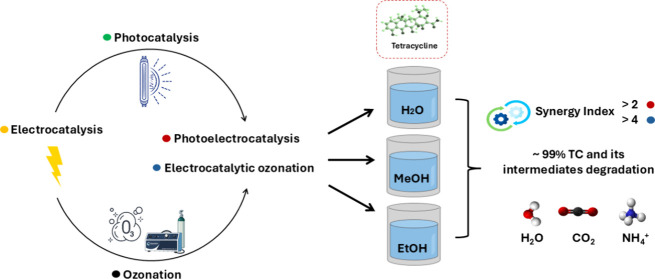

## 1 Introduction

Over recent decades, considerable efforts have focused on the remediation of priority pollutants such as polychlorinated biphenyls, heavy metals, hydrocarbons, and organochlorine pesticides.^1,2^ Advances in analytical techniques and the growing use of synthetic chemicals have also revealed the widespread occurrence of emerging contaminants (ECs) in aquatic environments, often at trace concentrations.^3,4^ Among these, antibiotics are of particular concern due to their extensive use and their role in driving antimicrobial resistance.^5^

Tetracycline (TC) is one of the most frequently detected antibiotics in surface water and wastewater, resulting from its broad application in human and veterinary medicine, agriculture, and animal husbandry.^2,6^ While effective as a bacteriostatic agent, its intensive use, combined with high chemical stability, contributes to its persistence in the environment.^7,8^ Even at low levels, TC can promote antibiotic resistance and pose toxicological risks to aquatic organisms and humans. Conventional wastewater treatment facilities often fail to fully remove TC, leading to its continuous discharge and accumulation in receiving waters.^9–12^

Traditional treatment methods for recalcitrant pollutants, including adsorption, coagulation–flocculation, membrane filtration, chemical precipitation, and ion exchange, have been widely studied.^2,13–16^ However, these approaches typically involve high costs, sludge generation, or incomplete removal.^13,17^ To overcome these limitations, advanced oxidation processes (AOPs) have emerged as effective alternatives. These methods, which include photocatalysis, electrochemical oxidation, ozonation, sonolysis, and their hybrid combinations, rely on the in situ production of reactive oxygen species (ROS) capable of mineralizing organic contaminants into harmless end products.^18–23^

Among single AOPs, ozonation is particularly attractive due to its strong oxidative potential and broad applicability, yet its efficiency is often limited by slow reaction kinetics and mass transfer constraints.^24,25^ To address these issues, hybrid processes have been developed. Photoelectrocatalysis (PEC) enhances charge separation in semiconductors under light irradiation, thereby improving ROS generation, while electrocatalytic ozonation (ECO) couples anodic reactions with ozone decomposition, producing abundant hydroxyl radicals. These combined approaches deliver higher degradation rates, improved mineralization, and reduced byproduct formation compared with their individual counterparts.^25–28^

Despite their promise, the large-scale application of AOPs depends strongly on electrode materials and process economics.^29,30^ Boron-doped diamond (BDD) electrodes remain the benchmark due to their high efficiency but are limited by prohibitive costs.^31,32^ Dimensionally stable anodes (DSA-Cl_2_), based on mixed metal oxides (MMOs), offer a cost-effective alternative with robust catalytic activity and durability, particularly in chloride-containing environments.^33–35^

Dimensionally stable anodes, composed of mixed metal oxides (MMOs) such as Ti/RuO_2_ and Ti/IrO_2_, have emerged as a cornerstone in AOPs due to their exceptional electrocatalytic activity, mechanical robustness, and relatively low cost compared to noble metals or BDD electrodes. Unlike conventional anodes that may suffer from chemical dissolution or passivating layer formation, MMO electrodes provide a stable substrate with a high surface area and tailored selectivity for specific oxidation reactions. These materials are particularly valued for their ability to catalyze the chlorine evolution reaction in chloride-rich environments, leading to the in situ generation of active chlorine species that significantly enhance the degradation kinetics of recalcitrant organic pollutants.^33^

The performance of MMO anodes is fundamentally linked to their surface composition and morphology, which facilitate efficient electron transfer and the generation of ROS. While BDD is often cited for its wide electrochemical potential window and high production of hydroxyl radicals, MMO electrodes offer a more economically viable and industrially scalable alternative with high current efficiency. Furthermore, the synergistic combination of different metal oxides (such as Ti, Ru, Ir, etc.) allows for the tuning of the overpotential for oxygen or chlorine evolution, making these anodes highly versatile for various wastewater treatment scenarios, including those involving complex organic matrices.^33–35^

Furthermore, the practical implementation of electrochemical treatment technologies can be significantly enhanced by decentralized wastewater management strategies, which offer more concentrated and less compositionally variable influences. Pretreatment methodologies, such as solvent extraction or adsorption, can facilitate the separation and concentration of contaminants, reducing the overall volume requiring treatment and improving process economics.^36,37^ Notably, the incorporation of an initial adsorption step followed by desorption with organic solvents (ethanol or methanol) can yield enriched TC solutions, favoring the formation of additional reactive species during AOPs. In fact, the presence of lower-polarity media and the partial oxidation of ethanol or methanol can generate reactive intermediates that potentiate oxidative pathways, ultimately improving degradation efficiency relative to purely aqueous systems.^38–41^

Accordingly, this work presents the application of three different advanced oxidation processes for the degradation of tetracycline in water, methanol, and ethanol. Throughout the study, detailed comparisons were made between the processes, analyzing their efficiencies in contaminant removal and their implications for energy consumption. Additionally, the synergistic effect when some of these processes are combined were calculated as well as the formation of degradation byproducts.

## 2 Materials and Methods

### 2.1 Chemicals

Tetracycline hydrochloride (C_22_H_24_N_2_O_8_, >95%) was acquired from Sigma-Aldrich. Methanol (99.9%) and ethanol (99.9%) were both obtained from Êxodo Cientifica, and hydrochloric acid (0.01 mol L^–1^) was used as a supporting electrolyte (37%—Synth). Methanol, HPLC grade (99.9%—Supelco), was used as a solvent and mobile phase in HPLC, along with acetonitrile, HPLC grade (99.98%—J.T. Baker), and oxalic acid P.A. (99.5–102.5%—Synth) 0.01 mol L^–1^. Deionized water (Millipore Milli-Q system, resistivity: 18.2 MΩ cm^–1^ at 25 °C) was used when necessary. All the chemicals were used as received without further purification.

### 2.2 Experimental Setup

The experiments were carried out using a one-compartment filter-press flow cell containing a titanium plate cathode and a DSA-Cl_2_ anode (Ti/Ti_0.7_Ru_0.3_O_2_), both with a geometric area of 14.81 cm^2^, separated by Viton insulators and Teflon spacers. The flow cell had a quartz window of 5 × 3 cm, which enabled the passage and incidence of UV–vis radiation through the solution and on the DSA-Cl_2_ surface for photocatalytic processes.

For the degradation experiments, a solution of 0.15 L of water, methanol, or ethanol with 0.01 mol L^–1^ HCl as a supporting electrolyte and 100 mg L^–1^ of TC was used at a flow rate of 500 mL min^–1^ controlled using a peristaltic pump (Solab—model SL-64). For the electrocatalytic processes, a current density of 5 mA cm^–2^ was applied using a power supply (Missipa, MPL-3303), and for the photocatalytic processes, a high-pressure Hg lamp (Philips) with a fluency rate of 0.113 W cm^–2^ was employed. The cell and lamp were located inside a closed box equipped with an exhaust fan to dissipate the heat produced by the irradiation. For the ozonation and electro-ozonation tests, an O&L 3.0-RM ozonator (Ozone & Life) was used, with an O_2_ flow rate of 0.5 L min^–1^ and an ozone generation rate of 50 μg mL^–1^. Prior to each experiment, the anode was polarized for 20 min at the current density of 40 mA cm^–2^ in a H_2_SO_4_ 0.5 mol L^–1^ solution to remove any impurities from the electrode.

### 2.3 Analytical Techniques

The TC concentration was monitored by high-performance liquid chromatography (HPLC—Shimadzu, LC-10AD VP) using a UV detector (Shimadzu, SPD-10A VP). As a stationary phase, a reversed-phase Agilent Zorbax SB-C18 column (5.0 μm, 250 mm × 4.6 mm) was implemented. The mobile phase was a mixture of methanol, acetonitrile, and oxalic acid (0.01 mol L^–1^), at isocratic mode, in a volumetric proportion of 40:5:55. A flow rate of 0.5 mL min^–1^, a detection wavelength of 360 nm, and an oven temperature of 25 °C were used.

The byproducts formed during TC degradation were identified through liquid chromatography–mass spectrometry (LC-MS). The analysis was performed on a Shimadzu liquid chromatograph equipped with a CBM-40 controller, two LC-40D XR binary pumps, a SIL-40C XR autoinjector, a PDA SPD-M40 detector (UV), and a CTO-40C oven, coupled to a Shimadzu LCMS-8045 triple quadrupole mass spectrometer (MS).

### 2.4 Toxicity and Bioaccumulation Prediction

The toxicity and bioaccumulation potential of tetracycline and its identified intermediates were estimated by using the Toxicity Estimation Software Tool (T.E.S.T.). The canonical SMILES of the parent compound and its byproducts were used as input for the software. Toxicity was evaluated through the lethal concentration 50 (LC_50_) for *Daphnia magna* (48 h exposure) and *Pimephales promelas* (fathead minnow, 96 h exposure). These values were calculated using the hierarchical clustering-based quantitative structure–activity relationship (QSAR) methodology, which predicts toxicity by identifying structurally similar compounds within the software training set. Additionally, the bioconcentration factor (BCF) was predicted to assess the bioaccumulation potential of aquatic organisms. The toxicity levels were categorized according to Clarkson index criteria.

## 3 Results and Discussion

Advanced oxidation technologies, including electrocatalysis, photocatalysis, ozonation, or their combinations, have emerged as promising alternatives for pollutant degradation, offering distinct mechanisms and efficiency advantages for the treatment of contaminated water. Comparing these processes and their efficiencies is essential to assess their large-scale feasibility, identify favorable operating conditions, and explore alternative strategies for effective wastewater treatment.^42^

### 3.1 Isolated Processes: Electrocatalysis (EC), Photocatalysis (PC), and Ozonation (Oz)

The degradation of TC in water, methanol, and ethanol was studied using a commercial electrode of mixed metal oxides. The implementation of these organic solvents, such as methanol and ethanol, stems from the previously mentioned preconcentration strategy, based on the use of activated carbon to adsorb the pollutant and finally desorb it with these solvents, to obtain a more concentrated solution with a smaller volume than the initial effluent. There are several studies showing the efficiency of using these solvents both to desorb contaminants retained in activated carbon and to oxidize them through electrochemical processes.^41,43–45^

Figure 1a shows the decay of TC concentration during electrocatalytic oxidation in different media. It was observed that in the first 10 min of electrolysis, methanol showed a faster decrease in TC concentration, while in water and ethanol, similar kinetic behaviors were observed, but with a slight difference in efficiency. This was reflected in the degradation percentages, achieving 99.9% TC removal in the methanol medium, 98.6% in water, and finally 92.5% in ethanol. The experimental data were fitted to a pseudo-first-order kinetic model. The validity of this model is demonstrated by the linear plots of ln­(*C*/C0) as a function of time, provided in Figures S1 and S2, with the calculated rate constants and their respective correlation coefficients summarized in Table S1.

**Figure 1:**
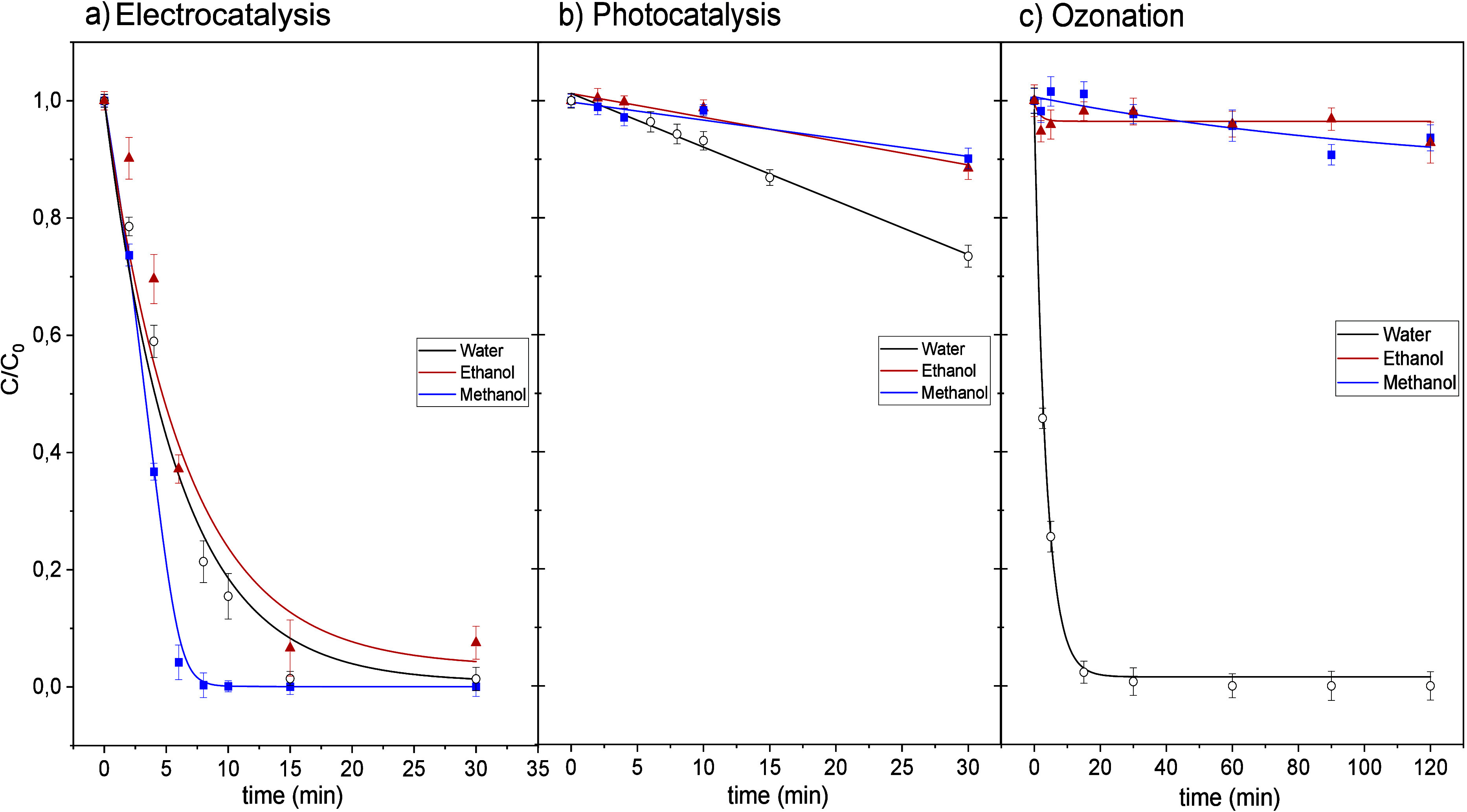
Degradation of TC by (a) electrocatalysis, (b) photocatalysis, and (c) ozonation; in (circle open) water, (box solid) methanol, and (triangle up solid) ethanol media (HCl = 0.01 mol L^–1^, *i* = 5 mA cm^–2^, O_2_ flow rate = 0.5 L min^–1^, O_3_ generation rate = 50 μg mL^–1^, lamp fluency rate = 0.113 W cm^–2^).

These results can be explained by the species formed in each medium. As widely mentioned in the literature, during electrooxidation in the aqueous medium, there can be the generation of different radicals and highly oxidizing species, where the nature of the anode will influence those species.^46,47^ The commercial MMO anode (Ti_0.7_Ru_0.3_O_2_) can efficiently degrade organic contaminants. RuO_2_ is widely used to manufacture these electrodes because it are resistant, offers advantages such as a reduced anode potential, durability, an electrochemically active area, and is known as a electrocatalyst in the formation of chlorine species.^48,49^

In the aqueous medium, due to the use of HCl as a supporting electrolyte, the presence of chloride ions can form highly reactive species, such as HClO, ClO^–^, and Cl_2_, capable of indirectly oxidizing the contaminant. Similarly, from the discharge of water on the electrode surface, the formation of hydroxyl radicals may occur, which can oxidize or also promote the formation of a species known as higher oxide (MO_*x*+1_) that can oxidize organics (eqs 1–3).^48,50,51^

MOx+H2O→MOx(OH●)+H++e− 1

MOx(OH●)→MOx+1+H++e− 2

$${\mathrm{MO}}_{x+1}+R\to {\mathrm{MO}}_{x}+\mathrm{RO}$$ 3

However, the use of an alcoholic medium during electrocatalysis largely suppresses the formation of these species. Previous studies using electron paramagnetic resonance spectroscopy showed that in methanol and ethanol media, alkoxy-type species such as ^●^OCH_3_ or ^●^OCH_2_CH_3_ are formed, as well as other minor species such as ^●^CH_2_OH, ^●^CH_2_CH_2_OH, and others. These radicals can also react with the pollutant and oxidize it efficiently, achieving considerably high TC removals.^52–54^ In terms of reactivity, the process is more efficient in methanol and water than in ethanol. In ethanol, the formation of two types of carbon-centered radicals (^●^CH_2_CH_2_OH or CH_3_(^●^CH)­OH) occurs via hydrogen abstraction from the carbon chain; these radicals are more stable and less reactive than the alkoxy-type radicals formed in methanol or the chlorine species and hydroxyl radicals generated in aqueous media.

In addition to reactive oxygen species, the presence of chloride ions in the electrolyte and the use of the Ti_0.7_Ru_0.3_O_2_ anode facilitate the chlorine evolution reaction (2Cl^–^ yields Cl_2_ + 2e^–^). At acidic pH, the chlorine gas hydrolyzes to form hypochlorous acid (HClO), a highly oxidizing species that can react with tetracycline through electrophilic substitution mechanisms. This pathway leads to the formation of halogenated disinfection byproducts, which must be carefully monitored because of their potential persistence and toxicity. This behavior has already been observed in the literature, where in an alcoholic medium there is a faster decay and degradation percentages close to those obtained in an aqueous medium.^54,55^

The implementation of UV–vis radiation has been a highly used strategy for degrading organic contaminants, especially with the implementation of photoactive catalysts that improve the efficiencies in pollutant removal.^56^ It has proven to be an effective technology in removing various compounds such as pesticides, dyes, and pharmaceuticals that are difficult to remove by conventional methods and without generating toxic byproducts during the degradation process. It offers advantages such as the possibility of being activated with visible light depending on the catalyst, which can be harnessed naturally (like sunlight), reducing the need for electrical energy for the process, making it more sustainable and economical. Moreover, photocatalysis can also help in the destruction of pathogenic microorganisms, such as bacteria and viruses.^57–59^

Figure 1b shows the decay of TC concentration during photocatalysis with the application of UV–vis radiation. It is possible to observe low efficiency for the three media, but in the aqueous medium, a slightly higher percentage was achieved, reaching a TC removal of 26.6%, while for methanol and ethanol, removals of 9.9 and 11.5% were obtained, respectively. Values of the pseudo-first-order kinetic constants are presented in Table S1.

This limited photocatalytic activity can primarily be attributed to the intrinsic physicochemical properties and electronic structure of Ti/RuO_2_ electrodes. One significant limitation is associated with the semiconductor characteristics of RuO_2_-based DSA electrodes. Typically, effective photocatalysts require suitable bandgap energies and appropriate band positions to generate reactive species (such as hydroxyl radicals (^•^OH)) through the formation of electron–hole pairs upon irradiation. However, RuO_2_ electrodes, as employed in this study, exhibit relatively narrow bandgaps and conduction band positions that are less favorable for efficient photocatalytic activity. Consequently, these materials can therefore suffer from rapid recombination of photogenerated electron–hole pairs, markedly decreasing the availability of reactive radicals required for pollutant degradation.^60^

Furthermore, RuO_2_-based DSAs, while highly effective as electrocatalysts due to their excellent conductivity, durability, and chlorine evolution reaction activity, do not generally exhibit strong adsorption affinity toward organic molecules like tetracycline. Low adsorption capacity limits direct interaction between the photocatalyst surface and the contaminant, reducing opportunities for degradation pathways initiated by surface-bound reactive species. Studies suggest that the formation of a thin rutile-type oxide film on Ti/RuO_2_ electrodes further reduces photoactivity, as this oxide configuration presents fewer active sites for pollutant interaction and degradation compared with traditional photocatalysts such as TiO_2_-based materials. Such surface passivation layers, formed during electrode preparation or pretreatment stages, hinder effective pollutant adsorption and electron-transfer processes.^61,62^

In the presence of solvents such as ethanol and methanol, competing solvent oxidation reactions may further diminish the photocatalytic performance. These organic solvents preferentially react with photogenerated reactive species, decreasing their availability for the targeted degradation of TC molecules. In contrast, although aqueous media yielded slightly higher photocatalytic performance, the overall low efficiency still emphasizes intrinsic photocatalyst limitations rather than solvent-dependent factors alone.^54,55^

A similar behavior was observed in nonaqueous media during ozonation of tetracycline (Figure 1c), where removal percentages were very low, i.e., only 6–7% in methanol and ethanol. In contrast, degradation in water was much more efficient, reaching 99.3% removal. This difference arises because, in aqueous media, ozone decomposes into highly reactive and nonselective hydroxyl radicals that rapidly attack tetracycline. In organic solvents like alcohols, the solvent competes with tetracycline for reaction with ozone and the generated radicals, consuming part of the oxidant before it can act on the pollutant. Moreover, water’s polarity and solubility properties enhance the stability, formation, and diffusion of reactive oxidative species, improving the interaction between tetracycline and these oxidants.^63–65^

### 3.2 Combined Processes: Photoelectrocatalysis (PEC) and Electrocatalytic Ozonation (ECOz)

Another methodology used due to its efficiency is photoelectrocatalysis based on the combination of photocatalytic and electrochemical processes to promote the degradation of organic contaminants. The combination of light with the application of electric current enhances catalytic reactions, making the process more efficient in removing complex and persistent organic pollutants by conventional methods, proving to be a promising alternative for the treatment of contaminated waters.^66–68^ Figure 2 shows how photoelectrocatalysis was efficient in the degradation of TC in both aqueous and organic media, achieving removals of 99.5, 99.4, and 99.3% for water, methanol, and ethanol, respectively. Values of the pseudo-first-order kinetic constants are presented in Table S1.

**Figure 2:**
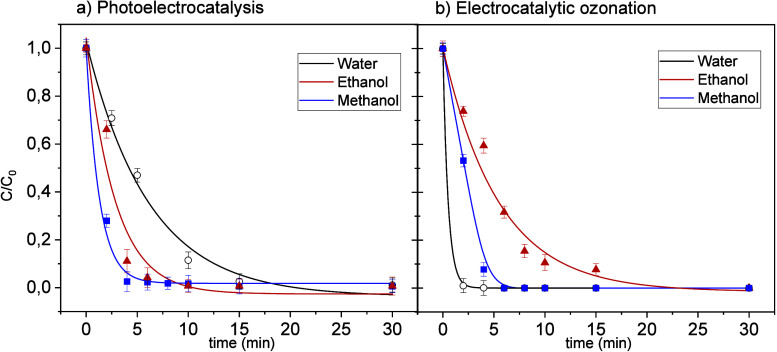
Degradation of TC by ((a) photoelectrocatalysis and (b) electrocatalytic ozonation; in (circle open) water, (box solid) methanol, and (triangle up solid) ethanol media (HCl = 0.01 mol L^–1^, *i* = 5 mA cm^–2^, O_2_ flow rate = 0.5 L min^–1^, O_3_ generation rate = 50 μg mL^–1^, lamp fluency rate = 0.113 W cm^–2^).

Several studies in the literature have also shown that both in the aqueous medium and in the methanol medium, the combination of these two processes has proven to be efficient in degrading organic contaminants.^69–73^ In the photoelectrocatalytic process, the simultaneous application of a current density along with UV-light irradiation has been shown to be complementary, mainly to prevent the recombination of charge carriers (eq 4), which increases the treatment efficiency.^66,67^

$${h_{\mathrm{BV}}}^{+}+{e_{\mathrm{BC}}}^{-}\to \mathrm{catalyst}+\mathrm{heat}$$ 4

Photoelectrocatalysis proved to be significantly better than the application of photocatalysis. However, when compared with electrocatalysis, similar behaviors and removal values were observed between these processes, indicating that the process by which the contaminant is oxidized is predominantly electrochemical. The efficiency of these processes is dependent on the applied current. At low current densities, electrocatalysis is less efficient than photocatalysis, but increasing the current substantially improves its performance, reaching removals similar to photoelectrocatalysis, albeit with higher energy consumption.^74^

On the other hand, the combination of electrocatalysis and ozonation resulted in high removal efficiencies within a short degradation time. Compared with ozonation, a significant improvement was observed in methanol and ethanol media, while a moderate enhancement was also noted when compared to electrocatalysis. Under these conditions, TC removal efficiencies of 99.9% were achieved in water and methanol after 4 and 6 min, respectively, whereas in ethanol, 30 min were required to reach the same removal percentages. This improvement is attributed to the synergistic effect between ozone and the electrochemical process, which promotes the generation of highly reactive oxidative species and enhances electron transfer at the electrode surface.^65^

Similar studies have also reported improvements when combining these two processes, demonstrating that the synergy between ozonation and electrocatalysis not only leads to higher degradation rates but also results in greater mineralization efficiency compared to the individual processes applied separately.^28,75^

The recent literature shows that photocatalysis utilizing materials like TiO_2_ decorated on natural pyrite achieved 100% TC removal but required 180 min of visible light irradiation.^4^ Other methods, such as the activation of persulfate by lotus leaf biochar (LLBC/PDS), reached an 86.58% degradation efficiency over 3 h. While aeration-assisted biochar processes can achieve up to 86.9% overall removal in 24 h, the actual degradation rate of the TC molecule itself remains relatively low (16.7%), with the process primarily relying on physical adsorption.^94^

Electrochemical and ozone-based systems have shown faster kinetics and higher mineralization rates. Electrochemical oxidation using BDD anodes achieved 100% chemical oxygen demand (COD) removal in approximately 50 min, though its maximum efficiency relies heavily on the generation of strong oxidizing agents in specific supporting electrolytes, such as NaCl.^88^ Conventional ozonation processes are also highly efficient, typically achieving complete removal within 40 to 120 min depending on the TC concentration and generation method.^9^ More recent cutting-edge technologies, such as ozone nanobubbles, have demonstrated 100% degradation of high concentrations of TC (100 mg L^–1^) in just 15 min due to enhanced mass transfer.^22^

Compared to these recent advancements, the ECOz process developed in our study presents a highly competitive and superior alternative. By synergistically combining electrochemical oxidation using a Ti_0.7_Ru_0.3_O_2_-MMO anode with continuous ozonation, our system achieved 99.9% degradation of a highly concentrated TC solution (100 mg L^–1^) in only 4 min in aqueous media. This coupled configuration exhibited a remarkable synergy index (SI = 4.76), rapidly accelerating reaction kinetics and promoting a deeper oxidation of intermediates. This comparative analysis (Table 1) justifies the importance of the proposed electro-ozonation system as a rapid, scalable, and highly efficient solution for antibiotic wastewater treatment.

**Table 1:** Comparison of Recent Technologies for the Degradation of Tetracycline (TC) in Aqueous Environments

method/process	catalyst/material	initial conditions	reaction time	degradation/removal efficiency	reference
**ozone nanobubbles**	none	TC: 100 mg L^–1^	20 min	100% degradation	^22^
		O_3_ flow: 10 L min^–1^			
**electrochemical oxidation**	boron-doped diamond (BDD) anode	TC: 100 mg L^–1^	10 min	100% TC degradation, 100% COD removal (in 30 min)	^88^
		electrolyte: 0.1 M NaCl			
**photocatalysis**	TiO_2_/natural pyrite (TiO_2_/NP)	TC: 30 mg L^–1^	180 min	100% TC removal	^4^
		catalyst: 2.0 g L^–1^		84% TOC removal	
**persulfate activation**	lotus leaf biochar (LLBC)	TC: 10 mg L^–1^	180 min	86.58% degradation	^94^
		PDS: 4 mM			
**aeration-assisted removal**	sewage sludge biochar (SSB)	TC: 10 mg L^–1^	24 h	86.7% total removal (16.7% direct degradation)	^101^
		aeration: 0.3 L min^–1^			
**UV-based AOP**	UV 222 nm/S_2_O_8_^2–^	TCH: 6.2 × 10^–5^ M	kinetic evaluation	high rate (*k* = 0.253 min^–1^)	^23^
**electro-ozonation**	Ti_0.7_Ru_0.3_O_2_ MMO/O_3_	TC: 100 mg L^–1^	4 min	>99 TC Removal Degradation	this study

The influence of the solvent matrix is strikingly evident when comparing the performance of individual versus coupled processes. The transition to integrated systems (PEC and ECOz) effectively overcomes solvent-induced limitations, maintaining removal efficiencies above 99% regardless of the matrix. This robustness demonstrates the synergy between electrochemical oxidation and UV or O_3_ pathways, which generates a higher density and diversity of reactive species, such as the solvent-derived radicals (alkoxy and carbon-centered types). Although the final removal percentage is high in all cases, the impact of the solvent remains detectable in the kinetic rate constants, which vary according to the solvent’s physicochemical properties. Therefore, coupled processes do not render the solvent irrelevant; instead, they provide the necessary oxidative power to overcome the inhibitory thresholds that typically hinder conventional advanced oxidation processes when applied separately.

### 3.3 Synergy Effect (SI) and Kinetic Constants (*K*)

When two processes are combined, the synergy index is an important parameter to be evaluated; it is a measure used to assess the interaction between two processes or factors involved in the system. It compares the system’s performance when the processes are applied simultaneously with the performance of each component separately, showing how the coupling of UV-light irradiation or ozonation along with the electrochemical processes complements and improves the efficiency of the process.

The synergy index is calculated by comparing the kinetic reaction rates (Table S1) in the photoelectrocatalysis and electrocatalytic ozonation systems with the sum of the reaction rates of the isolated processes (photocatalysis or ozonation and electrocatalysis). For this, the pseudo-first-order kinetic constants for each process were calculated and the values were applied in eqs 5 and 6:

$$\mathrm{synergy}\;{\mathrm{index}}_{\mathrm{PEC}}\;(\mathrm{SI})=\frac{{Κ}_{\mathrm{photoelectrocatalysis}}}{{Κ}_{\mathrm{electrocatalysis}}+{Κ}_{\mathrm{photocatalysis}}}$$ 5

$$\mathrm{synergy}\;{\mathrm{index}\;}_{\mathrm{EOz}}\;(\mathrm{SI})=\frac{{Κ}_{\mathrm{electrocatalytic}\;\mathrm{ozonation}}}{{Κ}_{\mathrm{electrocatalysis}}+{Κ}_{\mathrm{ozonation}}}$$ 6

It was observed that the kinetic constants of the photoelectrocatalytic process were greater than those of the separate processes, showing the synergistic effect between UV light and the electrochemical process, as reflected in the SI values obtained. In combined processes, when a synergy index of less than 1 is obtained, it indicates that the combination of photocatalytic and electrocatalytic effects is not synergistic or that there is no favorable interaction between these processes. In this case, the reaction rate does not improve, and the application of one process can actually reduce the effectiveness of the other, indicating an antagonistic behavior.

On the other hand, when the SI is equal to 1, it suggests that there is no synergy between the processes, as the photocatalytic and electrocatalytic effects are independent and the reaction rate in the photoelectrocatalytic system is merely the sum of the effects of the two isolated processes, without any significant improvement or loss due to the coupling of the processes. This behavior was observed during the photoelectrocatalytic degradation of TC in a methanol medium, obtaining an SI of 1.02; similar values have already been reported in previous research, where photoelectrocatalytic degradation in an organic medium did not show a synergistic effect, obtaining SI values close to 1.^73^

When water and ethanol were used as solvents, values of 1.61 and 2.43 were achieved, respectively. This indicates a positive synergistic effect between photocatalytic and electrocatalytic processes. The performance of the photoelectrocatalytic system is better than the sum of the isolated performances of each process (more significant in an ethanol than in an aqueous medium), which shows that the coupling of UV light and the application of a current density enhance the reaction rate, increasing the system’s performance. This synergy using MMO-type anodes in an aqueous medium has already been previously reported in the literature, showing an improvement in the process with the implementation of the combination of photocatalytic and electrocatalytic processes.^72,76^

On the other hand, the combination of ozonation and electrocatalysis processes has been shown in various studies to produce a synergistic effect that significantly enhances the efficiency of degrading complex pollutants. This synergy is observed through faster reaction rates, greater generation of reactive oxidative species, and more efficient ozone utilization. Then, during the photocatalytic ozonation, high removal percentages were achieved in nonaqueous media (methanol and ethanol), but no significant synergy effect between the processes was observed, with an SI close to 1 in both cases. In contrast, a value of 4.76 was obtained in aqueous media, indicating a strong synergy between ozone and electrocatalysis, even greater than that observed for photoelectrocatalysis. This marked difference highlights the critical role of the reaction medium in the overall efficiency of the combined process and supports previous findings in the literature, where the synergy between electrochemical methods and ozonation has led to substantial improvements in pollutant removal.^28,77,78^

### 3.4 Energy Consumption

Although the combined processes system has shown synergy and high removal efficiencies, it is necessary to evaluate which one presents a lower energy consumption. For this, eqs 7–11 were used, where *i* = applied current (A), *t* = electrolysis time or irradiation time (h), *V* = solution volume (m^3^), *E* = cell potential, and *W* = UV lamp or ozonizer power (W).^70,79,80^

$${\mathrm{EC}}_{\mathrm{electrocatalysis}}\;(\mathrm{Wh}m^{-3}{\%}^{-1})=\frac{i(A)\times t(h)\times E(V)}{V(m^{3})\times \mathrm{removal}(\%)}$$ 7

$${\mathrm{EC}}_{\mathrm{photocatalysis}}\;(\mathrm{Wh}m^{-3}{\%}^{-1})=\frac{W_{\mathrm{lamp}}(W)\times t(h)}{V(m^{3})\times \mathrm{removal}(\%)}$$ 8

$${\mathrm{EC}}_{\mathrm{ozonation}}\;(\mathrm{Wh}m^{-3}{\%}^{-1})=\frac{W_{\mathrm{ozonizer}}(W)\times t(h)}{V(m^{3})\times \mathrm{removal}(\%)}$$ 9

$${\mathrm{EC}}_{\mathrm{photoelectrocatalysis}}\;(\mathrm{Wh}m^{-3}{\%}^{-1})=\frac{\left[i(A)\times E(V)+W_{\mathrm{lamp}}(W)]\times t(h\right)}{V(m^{3})\times \mathrm{removal}(\%)}$$ 10

$${\mathrm{EC}}_{\mathrm{electrocatalytic}\;\mathrm{ozonation}}\;(\mathrm{Wh}m^{-3}{\%}^{-1})=\frac{\left[i(A)\times E(V)+W_{\mathrm{ozonizer}}(W)]\times t(h\right)}{V(m^{3})\times \mathrm{removal}(\%)}$$ 11

Figure 3 shows the energy consumption values required to achieve at least 90% of TC removal in water, methanol, and ethanol media for each process. Two types of energy consumption were represented: overall energy consumption (OEC) and effective energy consumption (EEC). The OEC was calculated considering only the total power consumption of the UV lamp and the ozone generator. In contrast, the EEC is based on the energy supply directly in the system; the UV lamp radiation with a fluency rate of 0.113 W cm^–2^, and the real concentration of ozone generated by the commercial generator, using a conversion factor of 0.26 under the conditions employed.^73,81–83^

**Figure 3:**
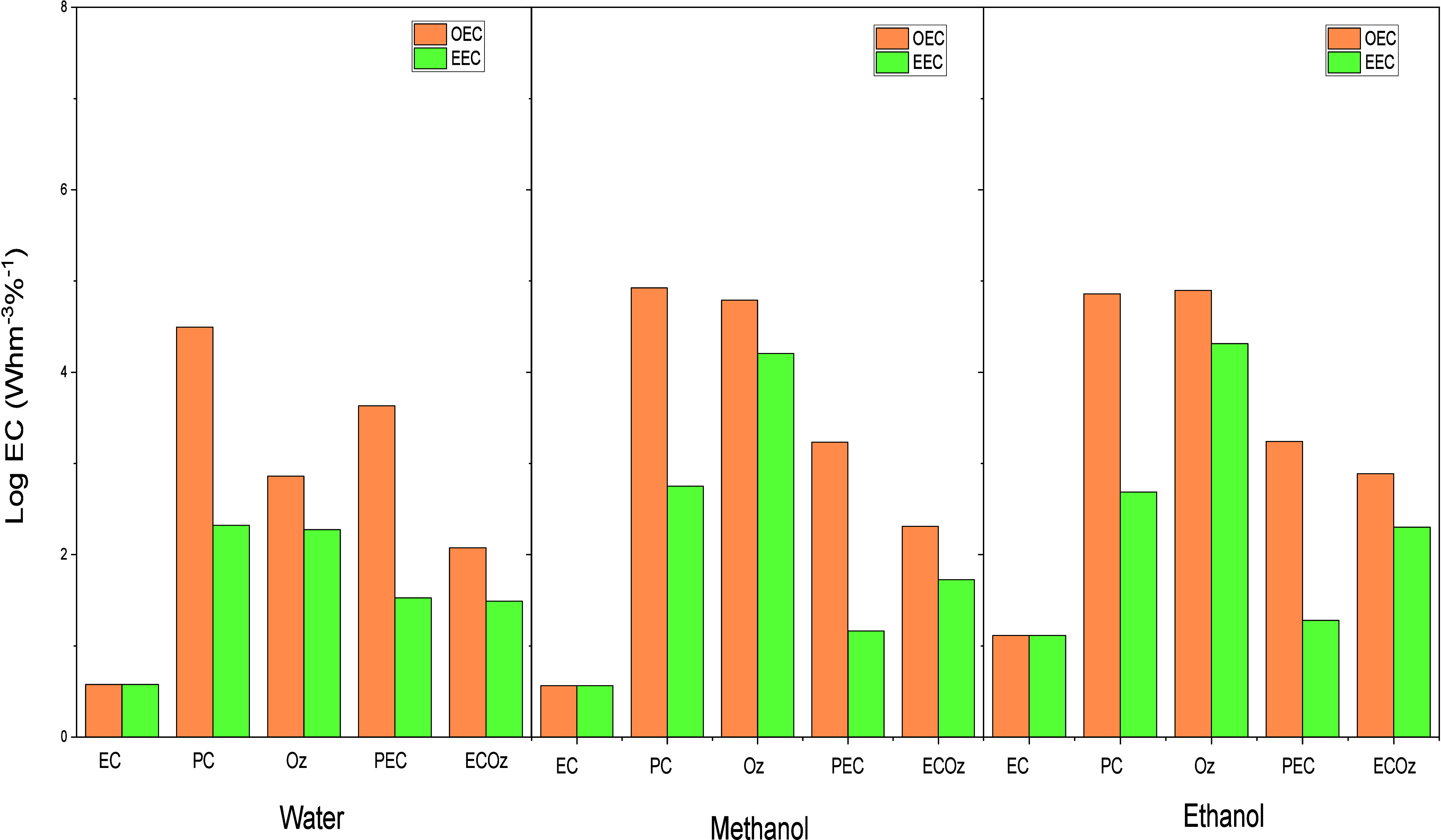
Overall energy consumption (OEC) and effective energy consumption (EEC) values for each process in water, methanol, and ethanol media (EC: electrocatalysis, P: photocatalysis, Oz: ozonation, PEC: photoelectrocatalysis, ECOz: electrocatalytic ozonation).

In general, the highest OEC was obtained by using photocatalysis and ozonation because both the UV lamp and the ozone generator required high powers for their operation, but by normalizing these values for the OEC of each process, these consumption values are lower. Although similar EECs were obtained for these two processes in aqueous media, using methanol and ethanol as solvents, consumption increased considerably when using ozonation because the TC degradation percentages were lower than those obtained with water.

In the electrocatalysis in water and methanol media, similar EEC values and degradation percentages were obtained. On the other hand, the ethanol medium showed an increase in EEC due to its high cell potential values. Due to its lower dielectric constant and electrical conductivity, there is a greater resistance of the solution, hindering the flow of current in the electrochemical system^72,73^ and still achieving slightly lower removals than the other media after 30 min of degradation. As previously mentioned, these values can be attributed to the higher resistance of the solution and the more reactive species formed in solution. Furthermore, ethanol also presents some parallel reactions that diminish TC degradation efficiency.^41,54,84–86^

For photoelectrocatalysis and electrocatalytic ozonation, lower EECs were achieved than in separate photocatalysis and ozonation, obtaining higher removal percentages in shorter time, and even when compared to electrocatalysis (mainly in water and methanol medium), relatively close EECs were reached compared to the others. Although the EEC for electrocatalysis was lower, there are also alternative strategies to improve the energy efficiency in the other systems. For instance, the implementation of advanced technologies to improve ozone generation efficiency in either commercial or custom-built cells can further contribute to reducing the overall energy demand of the process. Additionally, the integration of light-emitting diodes (LEDs) in photoelectrocatalytic processes represents a significant opportunity to reduce energy consumption while maintaining or even enhancing degradation efficiency. LEDs can consume up to 70% less electrical energy compared to traditional UV lamps, as demonstrated in studies where UV-LEDs achieved similar pollutant degradation rates with significantly lower energy input.^87^

### 3.5 Intermediates and Pathway of the TC Oxidation

In the present study, samples were collected at different time points, and intermediates were tentatively identified by comparing their total ion chromatograms. The structural assignments were based on *m*/*z* ratios and fragmentation patterns, following the established literature for tetracycline oxidation. These proposed pathways are presented as plausible degradation routes consistent with the experimental mass data. The M–H^–^ peaks of the initial tetracycline solution appeared at *m*/*z* 445. A diverse range of byproducts with varying intensities was detected, showing distinctions between each process and medium. Many intermediates were transient, completely disappearing by the reaction’s end.

Figure 4 illustrates degradation pathways during the photoelectrocatalytic process for the three media studied. In methanol-based experiments, partially oxidized products were identified, likely resulting from successive oxygen additions triggered by hydroxyl radicals (^•^OH) and other reactive species (e.g., radicals from methanol itself), evidenced by peaks at *m*/*z* 579 and 509.^88,89^ Some chlorinated intermediates were detected during the degradation process, which is directly related to the use of HCl as the supporting electrolyte. This provides a source of chloride ions that are oxidized at the DSA-Cl_2_ anode surface. Specifically, this electrode promotes the generation of active chlorine species. These species react with the tetracycline molecule through electrophilic substitution, leading to the formation of chlorinated byproducts. However, these chlorinated species act as transient intermediates and they are subsequently degraded and mineralized into simpler, less toxic fragments as the reaction progresses. Other significant intermediates observed during the reaction included *m*/*z* 435,^90,91^ 461,^92^ and 509.^93^

**Figure 4:**
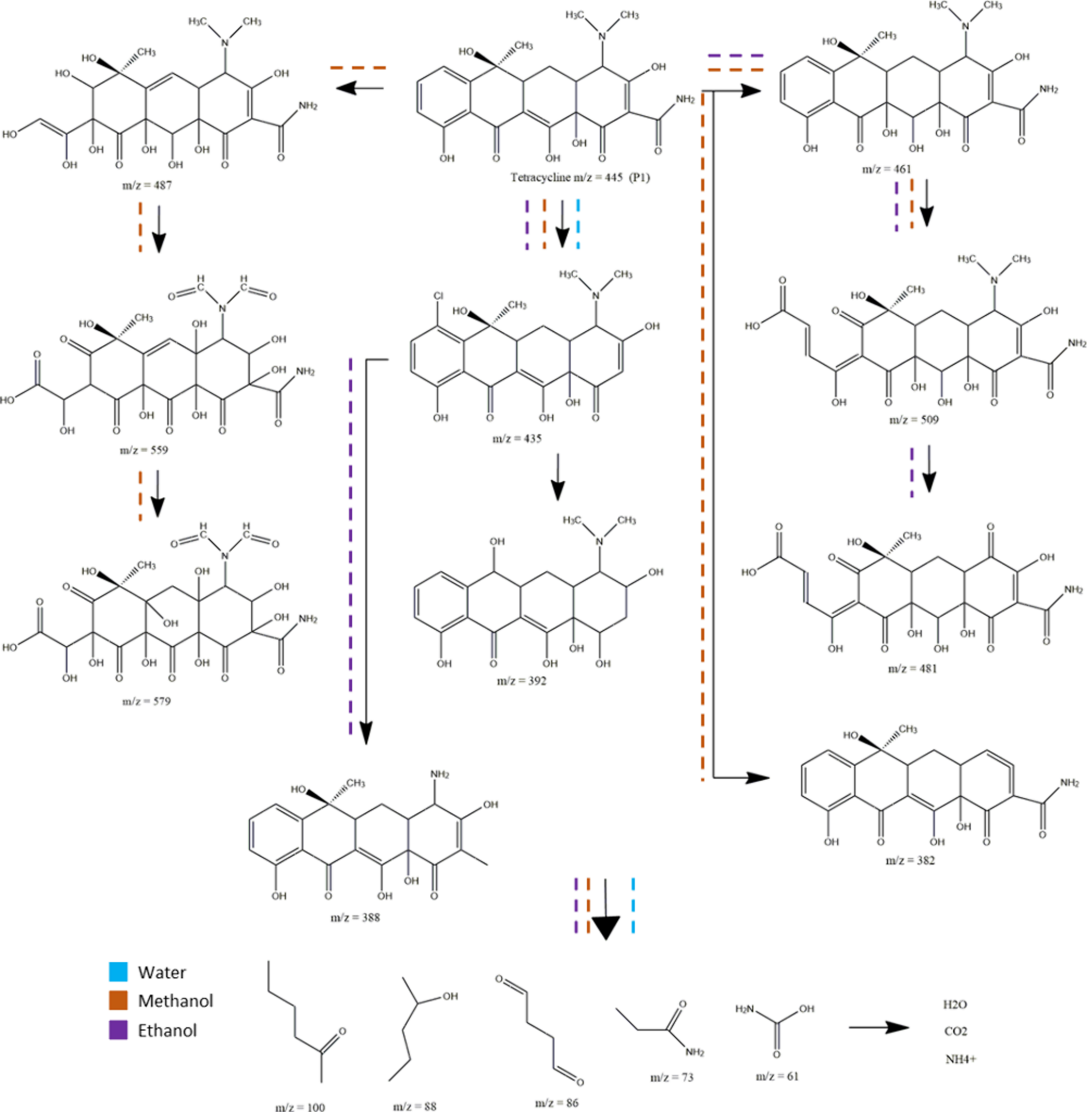
Intermediates of TC during the photoelectrocatalytic degradation.

For reactions in ethanol, significant intermediates appeared at *m*/*z* 435,^90,91^ 388,^82^ and 481.^82,83^ In aqueous reactions, only the peak at *m*/*z* 435 was prominent. Toward the reaction’s conclusion, all media exhibited residual peaks at *m*/*z* 100, 88, 86, 73, and 61, which completely disappeared by the end of the reaction.^94,95^

In the electrocatalytic process (Figure 5), experiments in methanol revealed peaks at *m*/*z* 435,^90,91^ 392,^96^ 461,^92^ 509,^93^ and 481.^95^ Ethanol experiments showed previously mentioned peaks plus additional peaks at *m*/*z* 579 and 387.^92^ The addition of a chlorine atom to the tetracycline aromatic ring explains the observed mass increases. In the pure electrocatalysis process, these compounds tend to be more persistent because of the structural stability provided by halogenation. Conversely, in ECOz systems, the synergy with ozone promotes the cleavage of these halogenated rings, explaining the lower detection of these peaks at the end of the treatment. The aqueous reactions presented peaks like methanol-based reactions with an additional peak at *m*/*z* 372.^96^ All peaks diminished by the end of the reaction, leading to the formation of peaks at *m*/*z* 100, 88, 86, 73, and 61, which disappeared by the end, indicating TC degradation through dehydration, demethylation, and hydroxylation processes.^97^

**Figure 5:**
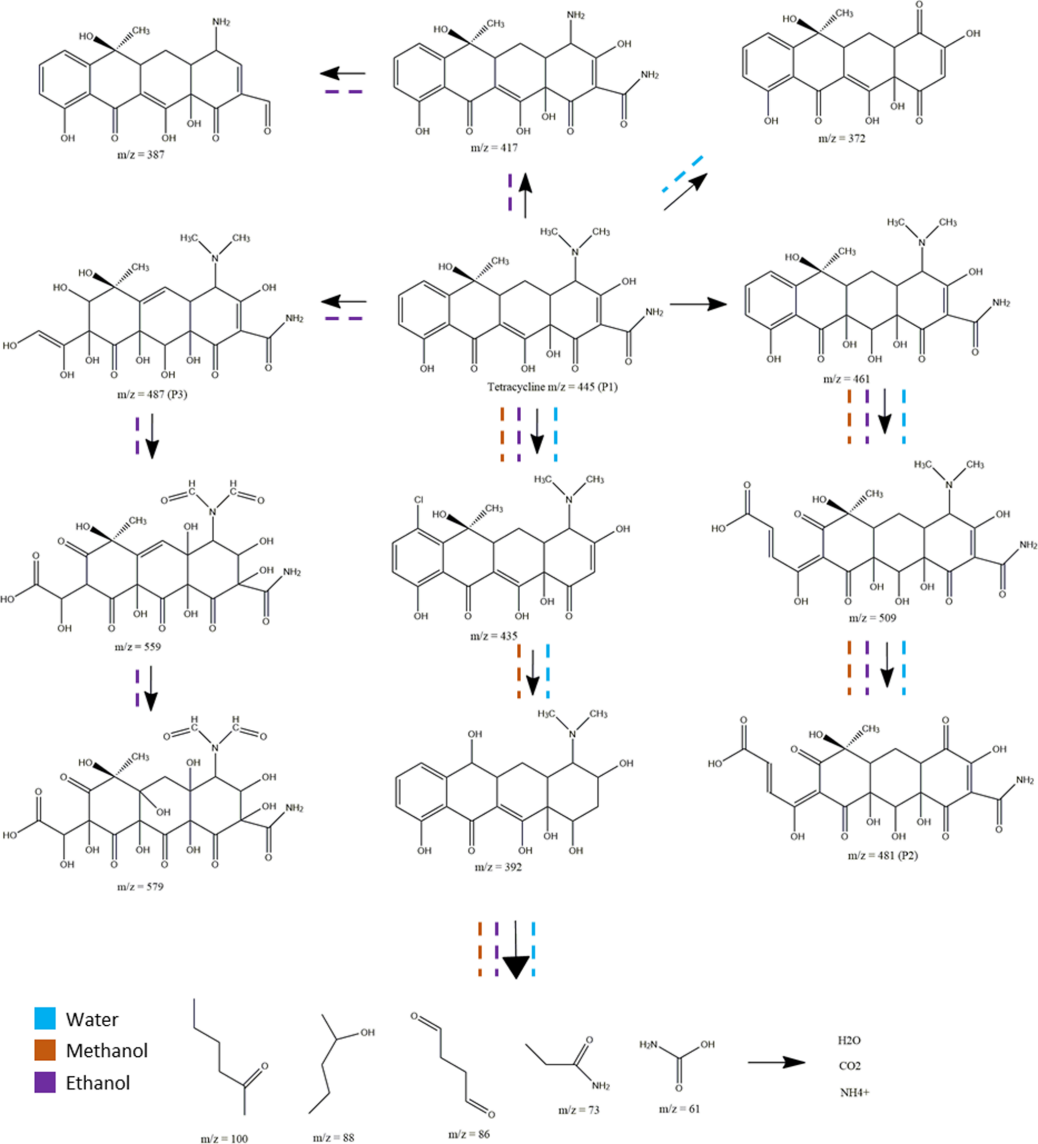
Intermediates of TC during the electrocatalytic degradation.

In the aqueous reaction, two subproducts emerged and persisted to the reaction’s conclusion: *m*/*z* 481 (P2) and 487 (P3).^98^ These compounds likely persisted due to increased structural stability acquired in initial oxidation stages, possibly involving aromatic arrangements or highly oxygenated structures with resonance, making them less susceptible to further radical attacks, such as ^•^OH.

In photocatalytic reactions (Figure 6), the *m*/*z* peaks at 579, 461, and 436 were consistent with those observed in other processes. Additional peaks were detected: in water and ethanol, *m*/*z* 483 (P4),^83^ 479 (P5),^81^ and 579 (P6); and in methanol, *m*/*z* 477 (P7),^99^ along with residual TC, persisted through to the reaction end, suggesting limited efficiency in degrading TC and its byproducts. For P4, LC50 values were 81.55 and 171.83 mg L^–1^.

**Figure 6:**
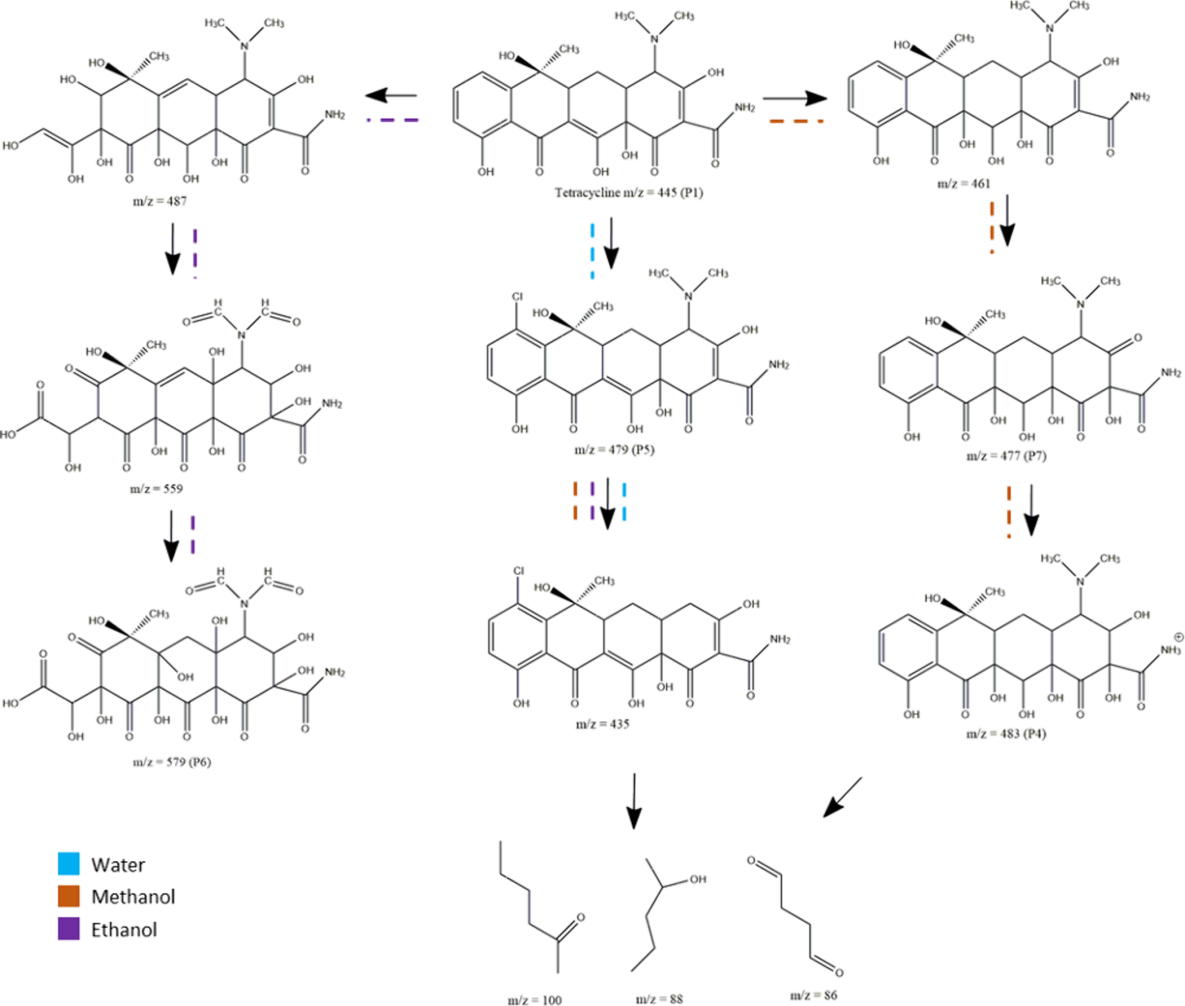
Intermediates of TC during the photocatalytic degradation.

In addition to the previously discussed methods, the degradation of tetracycline through an electrocatalytic ozonation process was also investigated to explore the alternative synergistic effects. The degradation pathway varied significantly with the solvent and the presence of an electrical current, as shown in Figure 7.

**Figure 7:**
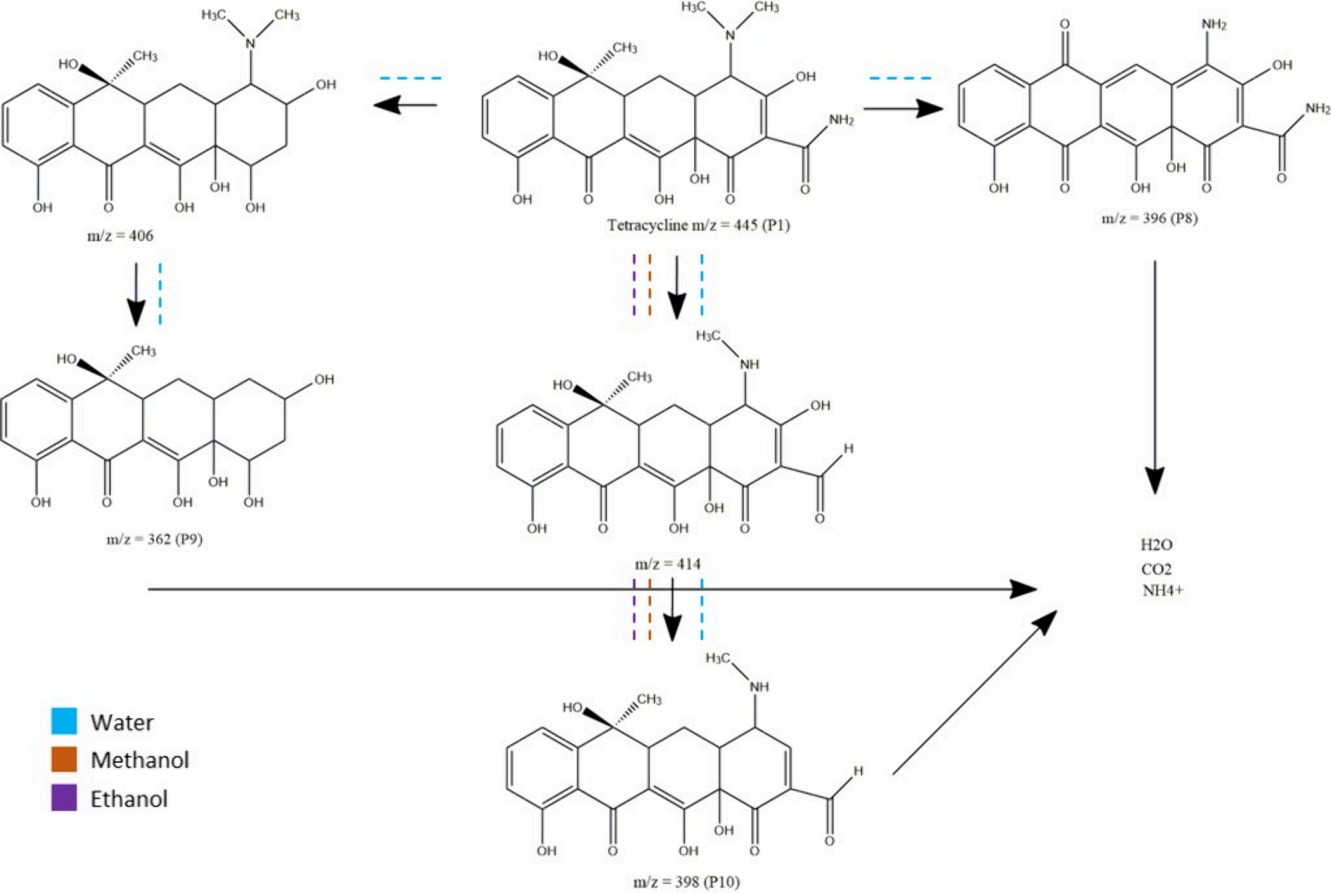
Intermediates of TC during the ozonation and electro-ozonation degradation.

In the electro-ozonation reactions conducted in methanol and ethanol, only one primary byproduct was formed, identified with an *m*/*z* of 398 (P10). The formation of this intermediate can be attributed to the cleavage of the dimethylamino group followed by the loss of a hydroxyl group from the tetracycline structure, a transformation consistent with previously reported photolytic degradation pathways where the naphthol ring remains intact.^100^

For aqueous ozonation reactions without electrochemical assistance, two main byproducts were observed at *m*/*z* 398 and *m*/*z* 396 (P8). The formation of the *m*/*z* 396 intermediate is well-documented and can occur through multiple routes, including the demethylation and oxidation of hydroxyl groups on the parent TC molecule^100,101^ or via dehydration of larger intermediates formed in earlier stages of oxidation.^102^

Interestingly, the combined electro-ozonation process in an aqueous medium yielded a distinct set of intermediates, including products with *m*/*z* 362 (P9) and a novel, previously unreported byproduct at *m*/*z* 573. The intermediate P3 (*m*/*z* 362) corresponds to a product formed after an initial ring cleavage to yield an intermediate of *m*/*z* 406, followed by deamination, a pathway that has also been observed in photocatalytic degradation studies.^103^ The novel intermediate at *m*/*z* 573 suggests a more complex degradation mechanism. The most plausible hypothesis is that this byproduct is an oxidative adduct, formed by the reaction of tetracycline (*m*/*z* 445) or one of its initial oxidation products with small, highly reactive fragments generated in situ. Ring-opening by ozonolysis is known to produce fragments such as aldehydes and carboxylic acids. In this case, tetracycline appears to undergo extensive oxidation, resulting in a net mass addition of 128 Da (likely corresponding to O_8_) to form the unstable intermediate. This massive oxidation likely involves the opening of one or more rings and the addition of multiple hydroxyl and carboxyl groups, which would explain its propensity for dehydration. Importantly, a common feature across all four ozonation and electro-ozonation conditions was the transient nature of these byproducts, which were degraded by the end of the treatment, underscoring the process’s high efficiency in removing not only the parent compound but also its transformation products.

In summary, the degradation of TC in the PEC and ECOz systems proceeds through a multistep mechanism initiated by the electrophilic attack of ROS and solvent-derived radicals. In aqueous media, hydroxyl radicals and ozone molecules preferentially attack the electron-rich sites of the TC molecule, such as the aromatic rings and the dimethylamine group, leading to hydroxylation and demethylation reactions. In the presence of organic solvents like methanol and ethanol, the mechanism is further mediated by alkoxy and carbon-centered radicals, which facilitate the cleavage of the C–C bonds. These initial transformations result in the formation of intermediates, which are subsequently oxidized toward complete mineralization.

The toxicity of TC and persistent intermediates, as well as their bioconcentration factors, was predicted using T.E.S.T. software. BCF values were employed to assess the accumulation potential of TC and its oxidation byproducts in aquatic organisms. BCF is defined as the concentration ratio of a compound in an organism relative to its concentration in the environment, indicating the bioaccumulation potential. Typically, compounds with BCF values below 1, such as TC (BCF = 0.71), exhibit moderate bioaccumulation potential, whereas values above 1 suggest greater likelihood of accumulation. The toxicity classification was determined based on the criteria established by Clarkson’s index, categorizing substances as toxic (LC50 values below 100 mg/L), moderately toxic (LC50 from 100 to 500 mg/L), slightly toxic (LC50 from 500 to 1000 mg/L), and nontoxic (LC50 above 1000 mg/L).

As presented in Table 2, the parent compound, tetracycline (P1), demonstrated significant toxicity, with LC50 values of 0.90 mg L^–1^ for fathead minnow and 5.44 mg L^–1^ for *Daphnia magna*. The degradation processes generated byproducts with distinct toxicity profiles. Some intermediates, such as P2 (*m*/*z* = 481), P5 (*m*/*z* = 479), and, most critically, P8 (*m*/*z* = 396), were found to be even more toxic than TC itself. Conversely, the formation of byproducts like P3 (*m*/*z* = 487) and P4 (*m*/*z* = 483) resulted in decreased toxicity compared to that of the parent compound. Regarding bioaccumulation, most oxidation byproducts exhibited BCF values (ranging from 1.03 to 8.14) notably higher than that of TC (0.71), indicating a greater potential for accumulation in aquatic organisms. Such elevated BCF values suggest that the structural modifications resulting from oxidation may have increased the lipophilicity and persistence of these molecules in the biological tissues. Only the byproduct P8 (*m*/*z* = 396) showed a BCF (0.47) lower than that of TC. The high toxicity of byproduct P2 (*m*/*z* 481) for *Pimephales promelas* (LC50 = 0.18 mg L^–1^) and *Daphnia magna* (LC50 = 1.71 mg L^–1^) corroborates its identification as a halogenated disinfection byproduct. Halogenated compounds are often more persistent and toxic than their original precursors, which reinforces the necessity of processes such as ECOz.

**Table 2:** Toxic Values of TC and Its Byproducts from T.E.S.T.

molecules	fathead minnow LC_50_(96 h - mg L^–1^)	*Daphnia magna* LC_50_(48 h - mg L^–1^)	bioconcentration factor
P1 (TC)	0.9	5.44	0.71
P2 (*m*/*z* = 481)	0.18	1.71	2.42
P3 (*m*/*z* = 487)	406.45	178.25	3.4
P4 (*m*/*z* = 483)	81.55	171.83	5.43
P5 (*m*/*z* = 479)	0.32	3.82	1.37
P6 (*m*/*z* = 579)	0.88	21.66	1.03
P7 (*m*/*z* = 477)	71.94	33.64	2.97
P8 (*m*/*z* = 396)	0.045	2.74	0.47
P9 (*m*/*z* = 362)	4.92	17.78	0.93
P10 (*m*/*z* = 398)	1.32	8.70	8.14

These findings represent significant advancements in degrading contaminants in alcoholic media from preconcentration processes, highlighting synergy among EAOPs. Contaminant degradation in alcoholic media was as efficient as that in aqueous media, and the synergistic effect of the photoelectrocatalytic mechanism effectively degraded the target contaminant and its intermediates, unlike separate processes.

## 4 Conclusions

Advanced oxidation technologies, including electrocatalysis, photocatalysis, and ozonation, demonstrated significant capabilities for degrading TC, although their effectiveness is highly dependent on the solvent matrix. Electrocatalysis alone achieved high removal efficiencies in all media, particularly in water and methanol (99.9%), while ozonation proved to be highly efficient in aqueous media (99.3%) but ineffective in organic solvents due to electron–hole recombination, poor pollutant adsorption, electrode passivation, and solvent competition.

While photoelectrocatalysis and electrocatalytic ozonation ensured removal rates exceeding 99% across all solvents, the analysis of the SI and EEC allows for a more rigorous technical recommendation. The significant synergy observed for photoelectrocatalysis in ethanol (SI = 2.43) and water (SI = 1.61) and for electrocatalytic ozonation in water (SI = 4.76) justifies the application of these integrated technologies in these specific matrices to accelerate degradation and manage persistent byproducts.

However, for the methanol medium, the synergy indices close to 1 indicate that combining processes offer no real catalytic advantage over the individual methods. In these cases, simple electrocatalysis is the most sustainable and energy-efficient option, as it achieves equivalent results without the additional energy demand required for UV lamp operation or ozone generation. Despite slightly higher energy consumption associated with UV irradiation or the ozone generator, these combined processes proved superior in promoting complete mineralization and the removal of intermediate byproducts, compared to the other processes separately.

In conclusion, the selection of the treatment process must be guided by the nature of the effluent and the necessity for full mineralization. While coupled systems are fundamental to ensuring environmental safety in matrices where intermediate persistence is a critical concern, the simplified application of electrocatalysis in specific organic matrices optimizes resources and economic viability of treating emerging contaminants.

## Supplementary Material



## Notes

Kinetic plots (Figures S1 and S2) of tetracycline degradation for all oxidation processes investigated in water, methanol, and ethanol media; (Table S1) corresponding pseudo-first-order kinetic constants (*k*) and correlation coefficients (*R* ^2^) for each system; and support of the analysis of reaction rates and the comparative performance of the different processes discussed in the manuscript (PDF)

## References

[1] Gheytanzadeh M., Baghban A., Habibzadeh S., Jabbour K., Esmaeili A., Mohaddespour A., Abida O. (2022). An insight into tetracycline photocatalytic
degradation by MOFs using the artificial intelligence technique. Sci. Rep..

[2] Nasiri A., Golestani N., Rajabi S., Hashemi M. (2024). Facile and green synthesis
of recyclable, environmentally friendly, chemically stable, and cost-effective
magnetic nanohybrid adsorbent for tetracycline adsorption. Helyion.

[3] Bashir Y., Raj R., Ghangrekar M. M., Nema A. K., Das S. (2023). Critical assessment
of advanced oxidation processes and bio-electrochemical integrated
systems for removing emerging contaminants from wastewater. RSC Sustainable.

[4] Hasham
Firooz M., Naderi A., Moradi M., Kalantary R. R. (2024). Enhanced
tetracycline degradation with TiO2/natural pyrite S-scheme photocatalyst. Sci. Rep..

[5] Bueno I., He H., Kinsley A. C., Ziemann S. J., Degn L. R., Nault A. J., Beaudoin A. L., Singer R. S., Wammer K. H., Arnold W. A. (2023). Biodegradation,
photolysis, and sorption of antibiotics in aquatic environments: A
scoping review. Sci. Total Environ..

[6] Mengting Z., Kurniawan T. A., Avtar R., Othman M. H. D., Ouyang T., Yujia H., Xueting Z., Setiadi T., Iswanto I. (2021). Applicability
of TiO_2_(B) nanosheets@hydrochar composites for adsorption
of tetracycline (TC) from contaminated water. J. Hazard. Mater..

[7] Xu L., Zhang H., Xiong P., Zhu Q., Liao C., Jiang G. (2021). Occurrence, fate, and risk assessment of typical tetracycline antibiotics
in the aquatic environment: A review. Sci. Total
Environ..

[8] Lin Y., Xu S., Li J. (2013). Fast and highly efficient tetracyclines removal from
environmental waters by graphene oxide functionalized magnetic particles. Chem. Eng. J..

[9] Amangelsin Y., Semenova Y., Dadar M., Aljofan M., Bjørklund G. (2023). The Impact
of Tetracycline Pollution on the Aquatic Environment and Removal Strategies. Antibiotics.

[10] Liu X., Zhang G., Liu Y., Lu S., Qin P., Guo X., Bi B., Wang L., Xi B., Wu F., Wang W., Zhang T. (2019). Occurrence and fate
of antibiotics
and antibiotic resistance genes in typical urban water of Beijing,
China. Environ. Pollut..

[11] Ahmad F., Zhu D., Sun J. (2021). Environmental
fate of tetracycline antibiotics: degradation
pathway mechanisms, challenges, and perspectives. Environ. Sci. Eur..

[12] Zheng H., Ji Y., Li S., Li W., Ma J., Niu J. (2023). Ecotoxicity
and resistance genes induction changing of antibiotic tetracycline
degradation products dominated by differential free radicals. Environ. Res..

[13] Nikzad M., Mousavi S. Y., Heydarian M., Rahmani S., Shabanian S. R., Hejazi F. (2024). A review on recent
advances in photodegradation of
tetracycline in aqueous media. J. Iran. Chem.
Soc..

[14] Esfandiaribayat M., Binazadeh M., Sabbaghi S., Mohammadi M., Ghaedi S., Rajabi H. (2024). Tetracycline removal from wastewater
via g-C3N4 loaded RSM-CCD-optimized hybrid photocatalytic membrane
reactor. Sci. Rep..

[15] Sivanesan J., Sivaprakash B., Rajamohan N., Phanindra V. S. S., Sonne C., Liew R. K., Lam S. S. (2024). Remediation
of tetracycline
pollution using microplastics, green materials, membranes and sonocatalysts:
a review. Environ. Chem. Lett..

[16] Jalloul G., Hijazi N., Boyadjian C., Awala H., Albadarin A. B., Ahmad M. N. (2024). Titania-zeolite
composite for tetracycline photocatalytic
degradation under visible light: A comparison between doping and ion
exchange. Heliyon.

[17] Antos J., Piosik M., Ginter-Kramarczyk D., Zembrzuska J., Kruszelnicka I. (2024). Tetracyclines contamination in European
aquatic environments:
A comprehensive review of occurrence, fate, and removal techniques. Chemosphere.

[18] Rabeie B., Mahmoodi N. M. (2024). Heterogeneous MIL-88A
on MIL-88B hybrid: A promising
eco-friendly hybrid from green synthesis to dual application (Adsorption
and photocatalysis) in tetracycline and dyes removal. J. Colloid Interface Sci..

[19] Sulaiman J. M. A., Altalbawy F. M. A., Kumar A., Kanjariya P., Rekha M. M., Siva
Prasad G. V., Singh D., Albadr R. J., Saydaxmetova S., Bhakuni P. N., athab A. H., Mansoor A. S., Radi U. K., Abd N. S., Muzammil K. (2025). Recent advances in
carbon nanomaterials: Removal, photodegradation and electrochemical
detection of tetracycline, a review. Inorg.
Chem. Commun..

[20] Niu J., Yuan R., Chen H., Zhou B., Luo S. (2024). Heterogeneous
catalytic ozonation for the removal of antibiotics in water: A review. Environ. Res..

[21] Samraj J. J., Manju R., Neppolian B. (2024). Sonochemical
degradation of tetracycline
and pharmaceutical effluent from the aqueous environment: Maximizing
efficiency with S-scheme photocatalyst. J. Water
Proc. Eng..

[22] Koundle P., Nirmalkar N., Momotko M., Makowiec S., Boczkaj G. (2024). Tetracycline
degradation for wastewater treatment based on ozone nanobubbles advanced
oxidation processes (AOPs) – Focus on nanobubbles formation,
degradation kinetics, mechanism and effects of water composition. Chem. Eng. J..

[23] Ishino A., Manyuan N., Kawasaki H. (2024). Degradation of Aqueous
Tetracycline
Hydrochloride through Radical-based Advanced Oxidation Processes Using
UV 222 nm/S2O82– and UV 222 nm/H2O2. J. Water Environ. Technol..

[24] Maharaja P., Athithyan I., Karthiyayini C., Kameswari K. S. B. (2025). Evaluation
of ozonation and electro oxidation treatment for the removal of organics
and salt recovery from RO reject from leather industries: Sustainable
approach for the management of contaminated salt in CETPs. Appl. Catal. O: Open.

[25] Chatfield E., Abbassi B. (2025). Evaluation of Electrocatalytic
Ozonation Process for
Hydroxyl Radical Production. Processes.

[26] G B., Banat F., Abu Haija M. (2023). Photoelectrochemical
advanced oxidation
processes for simultaneous removal of antibiotics and heavy metal
ions in wastewater using 2D-on-2D WS2@CoFe2O4 heteronanostructures. Environ. Pollut..

[27] Leng Q., Li F., Tao Z., Wang Z., Wu X. (2024). Advanced Wastewater
Treatment: Synergistic Integration of Reverse Electrodialysis with
Electrochemical Degradation Driven by Low-Grade Heat. Energies.

[28] Zhang H., Li S., Zhang C., Ren X., Zhou M. (2024). A critical review of
ozone-based electrochemical advanced oxidation processes for water
treatment: Fundamentals, stability evaluation, and application. Chemosphere.

[29] Zeng J., Xu R., El-Kady A. A., Oranj B. T., Ahmed R., Valentin R., Hu X., Wu W., Wang D., Mao J., Wu H., Gu X., Li P., Xu W., Zhang Z. (2023). Nanomaterials enabled
photoelectrocatalysis for removing pollutants in the environment and
food. TrAC - Trends Anal. Chem..

[30] Candia-Onfray C., Irikura K., Calzadilla W., Rojas S., Boldrin
Zanoni M. V., Salazar R. (2023). Degradation of contaminants of emerging
concern in a secondary effluent using synthesized MOF-derived photoanodes:
A comparative study between photo-, electro- and photoelectrocatalysis. Chemosphere.

[31] Clematis D., Panizza M. (2021). Application of boron-doped
diamond electrodes for electrochemical
oxidation of real wastewaters. Curr. Opin. Electrochem..

[32] Svítková J., Ignat T., Švorc L., Labuda J., Barek J. (2016). Chemical Modification
of Boron-Doped Diamond Electrodes for Applications to Biosensors and
Biosensing. Crit. Rev. Anal. Chem..

[33] Krstić V., Pešovski B. (2019). Reviews the
research on some dimensionally stable anodes
(DSA) based on titanium. Hydrometallurgy.

[34] A., Baciu ; S., Negrea ; F., Manea . Chapter 8: Electrocatalytic Degradation of Organic Pollutants from Water. In: Photocatalysts and Electrocatalysts in Water Remediation: From Fundamentals to Full Scale Applications; John Wiley & Sons Ltd., 2022, 241–273. 10.1002/9781119855347.ch8.

[35] Deng Z., Xu S., Liu C., Zhang X., Li M., Zhao Z. (2024). Stability
of dimensionally stable anode for chlorine evolution reaction. Nano Res..

[36] Rajan A., Sreedharan S., Babu V. (2016). Solvent Extraction
And Adsorption
Technique For The Treatment Of Pesticide Effluent. Civ. Eng. Urban Plan. Int. J..

[37] Coha M., Farinelli G., Tiraferri A., Minella M., Vione D. (2021). Advanced oxidation
processes in the removal of organic substances from produced water:
Potential, configurations, and research needs. Chem. Eng. J..

[38] Guo J., Chen R., Zhu F., Sun S., Villullas H. M. (2018). New understandings
of ethanol oxidation reaction mechanism on Pd/C and Pd2Ru/C catalysts
in alkaline direct ethanol fuel cells. Appl.
Catal. B: Environ..

[39] Santacruz W., Faria J., Nascimento O. R., Motheo A. J. (2025). Radical species
formation during electrochemical treatment of organic pollutants in
methanol: Effects of active and non-active anodes in chloride and
sulfate media. J. Electroanal. Chem..

[40] de
Mello R., Motheo A. J., Sáez C., Rodrigo M. A. (2022). Recent progress in the combination of activated carbon
adsorption and electrolysis for the treatment of waste. Curr. Opin. Electrochem..

[41] Norra G. F., Radjenovic J. (2021). Removal of persistent organic contaminants
from wastewater
using a hybrid electrochemical-granular activated carbon (GAC) system. J. Hazar. Mater..

[42] Valenzuela L., Villajos B., Mesa Medina S., Faraldos M. (2024). An Overview of the
Advantages of Combining Photo- and Electrooxidation Processes in Actual
Wastewater Treatment. Catalysts.

[43] Kim S., Kim Y. K. (2004). Apparent desorption kinetics of phenol in organic solvents
from spent activated carbon saturated with phenol. Chem. Eng. J..

[44] Muñoz-Morales M., Sáez C., Cañizares P., Rodrigo M. A. (2019). Enhanced electrolytic
treatment for the removal of clopyralid and lindane. Chemosphere.

[45] de
Mello R., Motheo A. J., Sáez C., Rodrigo M. A. (2022). Combination of granular activated carbon adsorption
and electrochemical oxidation processes in methanol medium for benzene
removal. Electrochim. Acta.

[46] Martínez-Huitle C. A., Andrade L. S. (2011). Electrocatalysis
In Wastewater Treatment: Recent Mechanism
Advances. Quim. Nova.

[47] Candido L., Gomes J. A. C. P. (2011). Evaluation
of anode materials for the electro-oxidation
of ammonia and ammonium ions. Mater. Chem. Phys..

[48] Duarte J. L. S., Meili L., Gomes L. M., Melo J. M. O., Ferro A. B., Tavares M. G., Tonholo J., Zanta C. L. P. S. (2019). Electrochemical
degradation of 17-α-Methyltestosterone over DSA® electrodes. Chem. Eng. Process: Process Intensif..

[49] Yu N., Lu X., Song F., Yao Y., Han E. (2021). Electrocatalytic degradation
of sulfamethazine on IrO2-RuO2 composite electrodes: influencing factors,
kinetics and modeling. J. Environ. Chem. Eng..

[50] Parra K., Gul S., Aquino J. M., Miwa D. W., Motheo A. J. (2016). Electrochemical
degradation of tetracycline in artificial urine medium. J. Solid State. Electrochem..

[51] Panizza M., Cerisola G. (2009). Direct And Mediated
Anodic Oxidation of Organic Pollutants. Chem.
Rev..

[52] Santacruz W., Faria J., De Mello R., Boldrin M. V., Motheo A. d. J. (2024). Comparative
study of MMO and BDD anodes for electrochemical degradation of diuron
in methanol medium. Chemosphere.

[53] Santacruz W., Fiori I., de Mello R., Motheo A. J. (2022). Detection of radicals
produced during electro-oxidation of atrazine using commercial DSA®-Cl2
in methanol media: Keys to understand the process. Chemosphere.

[54] Fiori I., Santacruz W., Dionisio D., Motheo A. J. (2022). Electro-oxidation
of tetracycline in ethanol-water mixture using DSA-Cl2 anode and stimulating/monitoring
the formation of organic radicals. Chemosphere.

[55] Faria J., Santacruz W., De Mello R., Boldrin M. V., Motheo A. J. (2024). Exploring
electrochemical mechanisms for clindamycin degradation targeted at
the efficient treatment of contaminated water. Chemosphere.

[56] Ahtasham
Iqbal M., Akram S., khalid S., Lal B., Hassan S. U., Ashraf R., Kezembayeva G., Mushtaq M., Chinibayeva N., Hosseini-Bandegharaei A. (2024). Advanced photocatalysis
as a viable and sustainable wastewater treatment process: A comprehensive
review. Environ. Res..

[57] Catanho M., Malpass G. R. P., Motheo A. J. (2006). Photoelectrochemical
treatment of
the dye reactive red 198 using DSA® electrodes. Appl. Catal. B: Environ..

[58] Zhang A., Wang W., Pei D., Yu H. (2016). Degradation of refractory
pollutants under solar light irradiation by a robust and self-protected
ZnO/CdS/TiO2 hybrid photocatalyst. Water Res..

[59] Ren G., Han H., Wang Y., Liu S., Zhao J., Meng X., Li Z. (2021). Recent Advances of
Photocatalytic Application in Water Treatment:
A Review. Nanomater. (Basel)..

[60] Tomaz A. T., Costa C. R., de Lourdes
S. Vasconcellos M., Pedicini R., Ribeiro J. (2022). Evaluation of Photoelectrocatalysis
with Electrode
Based on Ti/RuO2-TiO2Modified with Tin and Tantalum Oxides for the
Degradation of Indigo Blue Dye. Nanomaterials.

[61] Hussain T., Shabbir M. T., Anwar M., Bahadar A., Shakir S. (2023). Performance
evaluation of direct Z-scheme based modified titania composite for
photocatalytic degradation of organic pollutants. Opt. Mater..

[62] Ho V. T. T., Chau D. H., Bui K. Q., Nguyen N. T. T., Tran T. K. N., Bach L. G., Truong S. N. (2022). A High-Performing Nanostructured
Ir Doped-TiO2 for Efficient Photocatalytic Degradation of Gaseous
Toluene. Inorganics.

[63] Wang Y., Zhang H., Zhang J., Lu C., Huang Q., Wu J., Liu F. (2011). Degradation of tetracycline
in aqueous media by ozonation
in an internal loop-lift reactor. J. Hazard.
Mater..

[64] Reisz E., von Sonntag C., Tekle-Röttering A., Naumov S., Schmidt W., Schmidt T. C. (2018). Reaction of 2-propanol
with ozone
in aqueous media. Water Res..

[65] Miklos D. B., Remy C., Jekel M., Linden K. G., Drewes J. E., Hübner U. (2018). Evaluation
of advanced oxidation processes for water
and wastewater treatment – A critical review. Water Res..

[66] Sánchez-Montes I., Pérez J. F., Sáez C., Rodrigo M. A., Cañizares P., Aquino J. M. (2020). Assessing the performance of electrochemical oxidation
using DSA® and BDD anodes in the presence of UVC light. Chemosphere.

[67] Alulema-Pullupaxi P., Espinoza-Montero P. J., Sigcha-Pallo C., Vargas R., Fernández L., Peralta-Hernández J. M., Paz J. L. (2021). Fundamentals and
applications of photoelectrocatalysis as an efficient process to remove
pollutants from water: A review. Chemosphere.

[68] Zanoni M. V. B., Irikura K., Perini J. A. L., Bessegato G. G., Sandoval M. A., Salazar R. (2022). Recent achievements
in photoelectrocatalytic
degradation of pesticides. Curr. Opin. Electrochem..

[69] Hurwitz G., Pornwongthong P., Mahendra S., Hoek E. M. V. (2014). Degradation of
phenol by synergistic chlorine-enhanced photo-assisted electrochemical
oxidation. Chem. Eng. J..

[70] Zhu S., Dong B., Zhou S. (2018). Degradation
of Atenolol with Electrochemical
Oxidation at Mixed Metal Oxide Electrodes Assisted by UV Photolysis. Clean – Soil, Air, Water.

[71] Zhao Y., Fan Q., Wang X., Zhang W., Hu X., Liu C., Liang W. (2019). Photoelectrocatalytic degradation of microcystin-LR using a dimensionally
stable anode and the assessment of detoxification. Chem. Eng. J..

[72] Goulart L. A., Moratalla A., Lanza M. R. V., Sáez C., Rodrigo M. A. (2021). Photoelectrocatalytic treatment of levofloxacin using
Ti/MMO/ZnO electrode. Chemosphere.

[73] Santacruz W., de Mello R., Motheo A. J. (2023). New perspectives
to enhance the electro-oxidation
of atrazine in methanol medium: Photo assistance using UV irradiation. Chem. Eng. J..

[74] Malpass G. R. P., Miwa D. W., Miwa A. C. P., Machado S. A. S., Motheo A. J. (2009). Study of
photo-assisted electrochemical degradation of carbaryl at dimensionally
stable anodes (DSA®). J. Hazard. Mater..

[75] Yang D., Lv J., Shi Y., Gao B., Liu Z., Shao Z., Yao H., Li X. (2025). Three-dimensional electro-catalytic
ozonation for efficient
treatment of real urine wastewater: Synergistic effects and mechanism
investigation. Environ. Funct. Mater..

[76] Santos G. O. S., Eguiluz K. I. B., Salazar-Banda G. R., Saez C., Rodrigo M. A. (2020). Photoelectrolysis of clopyralid wastes
with a novel laser-prepared MMO-RuO2TiO2 anode. Chemosphere.

[77] García-Morales M. A., Roa-Morales G., Barrera-Díaz C., Bilyeu B., Rodrigo M. A. (2013). Synergy
of electrochemical oxidation using boron-doped diamond (BDD) electrodes
and ozone (O3) in industrial wastewater treatment. Electrochem. Commun..

[78] Bernal-Martínez L. A., Barrera-Díaz C., Solís-Morelos C., Natividad R. (2010). Synergy of
electrochemical and ozonation processes in industrial wastewater treatment. Chem. Eng. J..

[79] Thiam A., Sirés I., Brillas E. (2015). Treatment of a mixture of food color
additives (E122, E124 and E129) in different water matrices by UVA
and solar photoelectro-Fenton. Water Res..

[80] Dionisio D., Santos L. H. E., Rodrigo M. A., Motheo A. J. (2020). Electro-oxidation
of methyl paraben on DSA®-Cl2: UV irradiation, mechanistic aspects
and energy consumption. Electrochim. Acta.

[81] Souza F. L., Aquino J. M., Miwa D. W., Rodrigo M. A., Motheo A. J. (2014). Photo-assisted
electrochemical degradation of the dimethyl phthalate ester on DSA®
electrode. J. Environ. Chem. Eng..

[82] Sgroi M., Snyder S. A., Roccaro P. (2021). Comparison
of AOPs at pilot scale:
Energy costs for micro-pollutants oxidation, disinfection by-products
formation and pathogens inactivation. Chemosphere.

[83] Graça C. A. L., Lima R. B., Pereira M. F. R., Silva A. M. T., Ferreira A. (2020). Intensification
of the ozone-water mass transfer in an oscillatory flow reactor with
innovative design of periodic constrictions: Optimization and application
in ozonation water treatment. Chem. Eng. J..

[84] Prego M., Cabeza O., Carballo E., Franjo C. F., Jimenez E. (2000). Measurement
and interpretation of the electrical conductivity of 1- alcohols from
273 to 333 K. J. Mol. Liq..

[85] Mohsen-Nia M., Amiri H., Jazi B. (2010). Dielectric
Constants of Water, Methanol,
Ethanol,Butanol and Acetone: Measurement and ComputationalStudy. J. Solution Chem..

[86] de
Mello R., Rodrigo M. A., Motheo A. J. (2021). Electro-oxidation
of tetracycline in methanol media on DSA®-Cl2. Chemosphere.

[87] Tapia-Tlatelpa T., Buscio V., Trull J., Sala V. (2020). Performance analysis
and methodology for replacing conventional lamps by optimized LED
arrays for photocatalytic processes. Chem. Eng.
Res. Des..

[88] Li J., Zhai Z., Zhang C., Chen B., Hu T., Song H., Yang Y., Lv C., Liu L., Jiang X., Huang N. (2025). Electrochemical Oxidation Mechanism
of Tetracycline in Various Supporting Electrolytes Based on a Boron-Doped
Diamond Anode. Phys. Status Solidi A.

[89] Choina J., Bagabas A., Fischer Ch., Flechsig G.-U., Kosslick H., Alshammari A., Schulz A. (2015). The influence of the textural properties
of ZnO nanoparticles on adsorption and photocatalytic remediation
of water from pharmaceuticals. Catal. Today.

[90] Morrison H., Olack G., Xiao C. (1991). Organic photochemistry.
93. Photochemical
and photophysical studies of tetracycline. J.
Am. Chem. Soc..

[91] Jin X., Xu H., Qiu S., Jia M., Wang F., Zhang A., Jiang X. (2017). Direct photolysis of
oxytetracycline: Influence of initial concentration,
pH and temperature. J. Photochem. Photobiol.
A: Chem..

[92] Krakkó D., Heieren B. T., Illés A., Kvamme K., Dóbé S., Záray G. (2022). (V)­UV degradation of the antibiotic tetracycline: Kinetics,
transformation products and pathway. Process
Saf. Environ. Prot..

[93] Khan M. H., Bae H., Jung J. (2010). Tetracycline degradation
by ozonation in the aqueous
phase: proposed degradation intermediates and pathway. J. Hazard. Mater..

[94] Xu J., Song J., Guo H., Wang L. (2024). Investigation of tetracycline
degradation by activating persulfate with lotus leaf biochar: source
of active substance and toxicity evaluation. Water Sci. Technol..

[95] Liu R., Li M., Chen J., Yin Y., Zhao W., Gong Z., Jin H., Liu Z. (2024). Enhanced Photocatalytic
Degradation of Tetracycline
by Magnetically Separable g-C3N4-Doped Magnetite@Titanium Dioxide
Heterostructured Photocatalyst. Water.

[96] Nie M., Li Y., He J., Xie C., Wu Z., Sun B., Zhang K., Kong L., Liu Ji. (2020). Degradation of tetracycline
in water using Fe3O4 nanospheres as Fenton-like catalysts: kinetics,
mechanisms and pathways. New J. Chem..

[97] Ni S., Fu Z., Li L., Ma M., Liu Y. (2022). Step-scheme heterojunction
g-C3N4/TiO2 for efficient photocatalytic degradation of tetracycline
hydrochloride under UV light. Colloids Surf.
A: Physicochem. Eng. Asp..

[98] Chen G., Zhao L., Dong Y. (2011). Oxidative
degradation kinetics and
products of chlortetracycline by manganese dioxide. J. Hazard. Mater..

[99] Dalmázio I., Almeida M. O., Augusti R., Alves T. M. A. (2007). Monitoring
the
degradation of tetracycline by ozone in aqueous medium via atmospheric
pressure ionization mass spectrometry. J. Am.
Soc. Mass. Spectrom..

[100] Jiao S., Zheng S., Yin D., Wang L., Chen L. (2008). Aqueous photolysis of tetracycline and toxicity of photolytic products
to luminescent bacteria. Chemosphere.

[101] Liu L., Lei K., Gao X., Zhang M., Wang H., Shan S., Joseph S. D., Fang J. (2025). Aeration-assisted removal
of tetracycline from wastewater by biochar: mechanisms and cost-benefit
analysis. Environ. Res..

[102] Wang J., Zhi D., Zhou H., He X., Zhang D. (2018). Evaluating tetracycline degradation pathway and intermediate
toxicity
during the electrochemical oxidation over a Ti/Ti4O7 anode. Water Res..

[103] Su F., Li P., Huang J., Gu M., Liu Z., Xu Y. (2021). Photocatalytic degradation of organic dye and tetracycline
by ternary
Ag2O/AgBr–CeO2 photocatalyst under visible-light irradiation. Sci. Rep..

