# Supplementary material for: Process Intensification of Tetracycline Degradation: Synergistic Electrocatalytic Ozonation and Photoelectrocatalysis in Aqueous and Organic Matrices
Source: ACS Omega. 2026 Jun 30;11(27):40729–44. doi: 10.1021/acsomega.6c04336 (PMC13382748; doi:10.1021/acsomega.6c04336)
Supplement: Supplementary file 1 [file ao6c04336_si_001.pdf]

Process Intensification of Tetracycline Degradation: Synergistic Electrocatalytic Ozonation and Photoelectrocatalysis in Aqueous and Organic Matrices

William Santacruz[a*], Michel Z. Fidelis[a], Julia Faria[a], Artur J. Motheo[a]

[a] São Carlos Institute of Chemistry, University of São Paulo (USP), São Carlos, SP, CEP 13560-97, Brazil.

* **Corresponding author**. E-mail: williamsp@usp.br

SUPPORTING INFORMATION

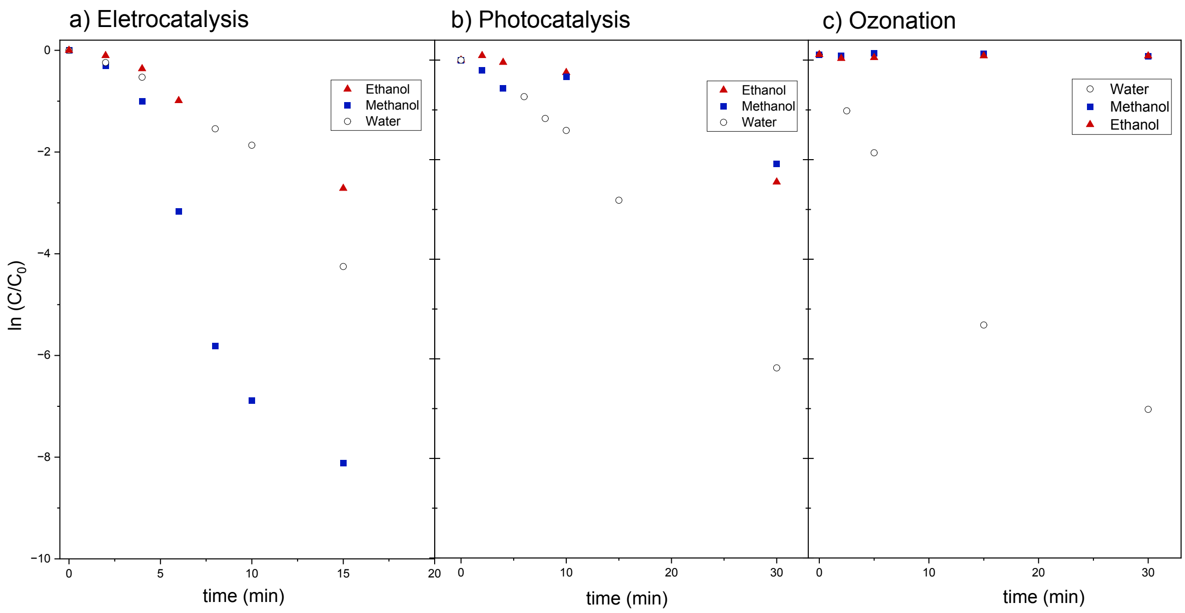

Figure S1. Kinetic plots of ln (C/C_0) vs. time for TC degradation by a) electrocatalysis, b) photocatalysis and c) ozonation; in (○) water (■) methanol, and (▲) ethanol medium (HCl= 0.01 mol L^{-1}, i= 5 mA cm^{-2}, O_2 flow rate= 0.5 L min^{-1}, O_3 generation rate= 50 µg mL^{-1}, Lamp fluency rate= 0.113 W cm^{-2}.)

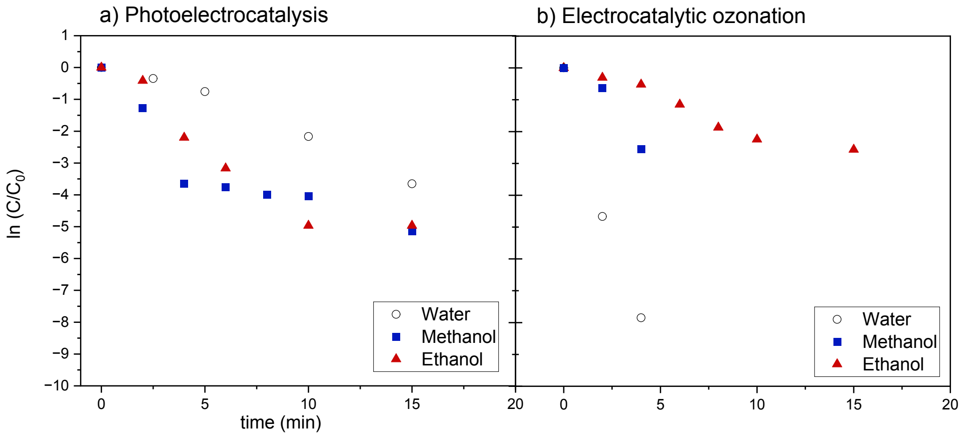

Figure S2. Kinetic plots of ln (C/C_0) vs. time for TC degradation by a) photoelectrocatalysis and c) electrocatalytic ozonation; in (○) water, (■) methanol, and (▲) ethanol medium (HCl= 0.01 mol L^{-1}, i= 5 mA cm^{-2}, O_2 flow rate= 0.5 L min^{-1}, O_3 generation rate= 50 µg mL^{-1}, Lamp fluency rate= 0.113 W cm^{-2}.)

Table S1. Values of pseudo-first-order kinetic constants

		**EC**	**PC**	**Oz**	**PEC**	**EOz**
**water**	k	**-0,17335** ± 0,0121	**-0,00986** ± 4,82123E-4	**-0,25442** ± 0,0066	**-0,20046** ± 0,0166	**-2,03629** ± 0,1049
	R^2	0,97623	0,99052	0,99798	0,97923	0,99472
**methanol**	k	**-0,58363** ± 0,09021	**-0,00335** ± 3,65932E-4	**-5,92511E-4** ± 7,20032E-5	**-0,59701** ± 0,0685	**-0,57422** ± 0,09151
	R^2	0,91277	0,95454	0,94422	0,94997	0,95166
**ethanol**	k	**-0,20998** ± 0,02313	**-8,38328E-4** ± 1,5393E-4	**-6,37927E-4** ± 3,3138E-5	**-0,51126** ± 0,04905	**-0,21361** ± 0,01288
	R^2	0,96489	0,90815	0,98932	0,97313	0,98215
